# Cardiovascular effects of standardized hydroalcoholic extract of *Ribes khorasanicum *fruit in acute hypertensive rats

**Published:** 2020

**Authors:** Ismael Hamounpeima, Reza Mohebbati, Mahmoud Hosseini, Abolfazl KhajaviRad, Hasan Rakhshandeh, Abbas Safarnejad, Mohammad Naser Shafei

**Affiliations:** 1 *Department of Physiology, Faculty of Medicine, Mashhad University of Medical Sciences, Mashhad, Iran*; 2 *Neurogenic Inflammation Research Center, Mashhad University of Medical Sciences, Mashhad, Iran*; 3 *Pharmacological Research Center of Medicinal Plants, School of Medicine, Mashhad University of Medical Sciences, Mashhad, Iran*; 4 *Faculty of Khorasan Razavi Agricultural and Natural Resources Research Center, Education and Extension Organization (AREEO), Mashhad, Iran*

**Keywords:** Ribes khorasanicum Angiotensin II, Blood pressure, Herat rate, Hypertension

## Abstract

**Objective:**

*Ribes khorasanicum* (*R. khorasanicum*) traditionally has been used for the treatment of higher blood pressure. In this study, the effect of hydroalcoholic extract of *R. khorasanicum* fruit in normotensive and hypertensive rats was evaluated.

**Materials and Methods:**

Animals were assigned into the following groups: 1) Control, 2) AngII (50 ng/kg), 3) AngII + losartan (Los, 10 mg/kg) and 4-6) Doses 4, 12 and 24 mg/kg of extract +AngII groups. AngII and Los were injected intravenously and the extract was injected intraperitoneal. In *R. khorasanicum* groups, AngII injected 30 after injection of the extract. The femoral artery was cannulated and mean arterial pressure (MAP), systolic blood pressure (SBP), and heart rate (HR) were recorded by Power Lab software. Maximal changes (∆) of cardiovascular responses were determined and compared with those of control and AngII groups. Finally, oxidative stress parameters in the heart and aorta were also determined.

**Results:**

In normotensive rats, 12 mg/kg of the extract showed significant hypotensive effects while 24 mg/kg produced significant tachycardia. Increased ∆SBP and ∆MAP in AngII group were significantly blunted by Los. Doses 4 and 12 mg/kg of the plant also significantly attenuate the effect of AngII on ∆SBP and ∆MAP. Tachycardia induced by 24 mg/kg of the extract didn't affect by AngII. Extract also significantly improved the effect of AngII on MDA, total thiol content, CAT and SOD in both heart and aorta tissues.

**Conclusion:**

*R. khorasanicum *at lower doses showed hypotensive effects and attenuated cardiovascular parameters in hypertensive rats via its antioxidant effects.

## Introduction

*Ribes khorasanicum (R. khorasanicum)* belongs to Grossulariaceae family grows in North Khorasan province of Iran, especially mountains of Hezarmasjed and the city of Dargaz. The local name of this plant is *Qareghat* that was identified by saghafi and asadi in 1996 (Saghafi et al., 1996 ▶). There is a little information about the components and curative effects of this plant. Adibi et al reported that fruit of *R. khorasanicum* contains compounds such as flavonoids, saponins, tannins, and alkaloids (Adibi et al., 2007 ▶). The presence of anthocyanin, protein, ascorbic acid also indicated in another study (Yazdi et al., 2018b ▶). Indigenous peoples and villagers use dried fruits of *R. khorasanicum* for the high blood pressure treatment, remedy gastrointestinal poisoning and heart disease (Adibi et al., 2007 ▶; Moradi et al., 2016 ▶). The antibacterial, antifungal and antioxidant effects of this plant were reported previously (Adibi et al., 2007 ▶; Yazdi et al., 2018a ▶).

The renin-angiotensin system (RAS) is involved in the regulation of several systems of the body such as physiological regulation of cardiovascular system. However, overproduction of RAS by the effect on numerous biological processes such as vasoconstriction, endothelial dysfunction, vascular stress oxidative and inflammation (Schmieder et al., 2007 ▶) improve the development and maintenance of hypertension. Also, anti-hypertension, anti-inflammatory, and antioxidant effects have been shown after blockade of RAS (Sata et al., 2010 ▶). Angiotensin II (AngII) is the well-known products of RAS that is produced from angiotensinogen in two phases: initially angiotensinogen by renin converted to angiotensin I, and then this product converts to AngII by the effect of angiotensin-converting enzyme (ACE). It has been shown that several cardiovascular effects of RAS such as vasoconstriction, endothelial dysfunction, vascular hypertrophy and oxidative stress are mediated by AngII and its AT1 receptor (Polizio et al., 2005 ▶; Sata et al., 2010 ▶).

The effect of medicinal plants on high blood pressure induced by RAS, especially AngII has been shown in previous studies (Enayatfard et al., 2018 ▶; Perez et al., 2010 ▶).

Previously, it was also indicated that the antihypertensive effect of *Teucrium polium* and crocin is mediated partly by the effect on AngII (Mahmoudabady et al., 2014 ▶; Shafei et al., 2017 ▶). It has also reported that AngII by increasing oxidative stress contribute in hypertension (Romero et al., 1999 ▶). Because *R. khorasanicum* has an antioxidant effect, it is possible that antihypertensive properties of this plant also are mediated by the antioxidant effect. Therefore, in the current study, the probable effects of hydroalcoholic extract fruit of *R. khorasanicum* on cardiovascular responses and oxidative stress in acute hypertension induced by AngII were investigated.

## Materials and Methods

**Plant and extract preparation**

Fruit of *R. khorasanicum* was collected from Dargaz (North Khorasan Province, Iran) and identified by botanists in the agricultural and Natural Resources Research Center of Mashhad (No:3242).

The hydroalcoholic extract was prepared by adding 100 g of dried powder of *R. khorasanicum* fruit to 1800 ml of ethanol 70% (540 ml distilled water and 1260 ml ethanol) and macerated for 72h with occasional shaking. Then, the extract was filtered and solvent was removed by a rotatory evaporator.

**Figure1:**
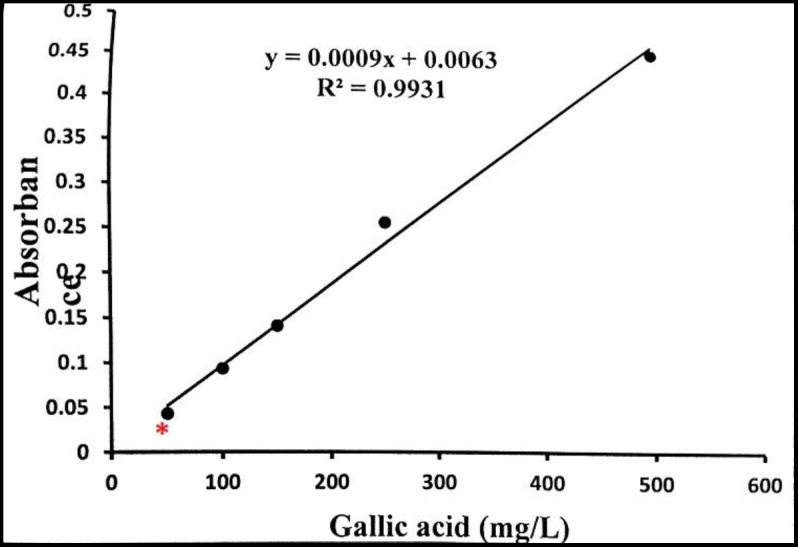
Standard curve of gallic acid. The asterisk (*) represents the amount of phenol in the extract.

For standardization of extract , the total phenol content was determined by Folin-Ciocalteu method (Ainsworth et al., 2007 ▶). Based on this method, the standard curve was provided for gallic acid and the total phenol concentration of the extract was expressed as milligram of gallic acid equivalent. The total phenol concentration in the *R. khorasanicum* extract was 42.4 mg gallic acid equivalent/g crude extract (Figure1). 

**Animals and surgery**

Sixty male Wistar rats (220-250 g) were used in this study. The animals were anesthetized with urethane (1.4 g/kg, i.p), and its supplementary dose (0.7 g/kg) (Shafei et al., 2012) and were kept at 37°C by a heating lamp. For recording of blood pressure, cannulation of left femoral artery was performed using a polyethylene catheter (PE-50) filled with heparinized saline. Femoral catheter then was attached to a blood pressure transducer. Cardiovascular parameters (systolic blood pressure (SBP), mean arterial pressure (MAP) and heart rate (HR)) were continuously recorded by a power lab apparatus (ID instrument, Australia) (Shafei et al., 2013 ▶). 

**Drug and animal groups**

AngII, urethane, and losartan (Los) were purchased from Sigma Chemical Company (USA, city). Solvent of All drugs was saline. AngII and Los were injected intravenously (i.v) via the left femoral vein and the extract injected intraperitoneal (i.p). 

Animals were divided into the following 6 groups: 

1. Control group; received saline (i.v)

2. AngII group; received AngII (50 ng/kg, i.v)

3. Los group; received Los (10 mg/kg, i.v) 

4. *R. khorasanicum* 4+AngII group; received 4 mg/kg of *R. khorasanicum* before AngII

5. *R. khorasanicum* 12+AngII group; received 12 mg/kg of *R. khorasanicum*) before AngII 

6. *R. khorasanicum* 24+AngII group; received 24 mg/kg of *R. khorasanicum* before AngII 

Doses of extract selected based on a pilot study.

**Experimental Protocol**

After stabilization time ( 20 min), the AngII group received AngII (50 ng/kg, i.v) (Brunner et al., 1972) intravenously. In AngII + Los group firstly, Losartan (10 mg/kg, i.v) (Croquet et al., 2002) and after 10 min AngII were injected and cardiovascular responses were recorded. In treated groups with *R. khorasanicum* extract, rats were received three doses (4, 12 and 24 mg/kg) of hydroalcoholic extract of *R. khorasanicum* intraperitoneal and 30 min after that changes of basal cardiovascular parameters were determined. After that AngII (50 ng/kg, i.v) was injected and maximum changes (∆) of MAP, SBP, and HR induced by AngII were obtained. 

**Oxidative stress assessment**

After recording of cardiovascular parameters, the animals were killed with a higher dose of anesthetic drugs. Then their heart and aorta tissues were removed. The samples were homogenized with PBS and oxidative stress factors including total thiol content (SH), malondialdehyde (MDA), catalase (CAT) and superoxide dismutase (SOD) were determined using commercial methods as it has been previously reported (Beheshti et al., 2019 ▶; Beheshti et al., 2018 ▶). 

**Data analysis**

The changes() of SBP, MAP and HR values were expressed as mean ± SEM. Statistical analysis performed by One-way ANOVA followed by the Tukey’s post hoc test. A value of p<0.05 was used to indicate statistical significance.

## Results

**Effects of three doses of** ***R. khorasanicum*** **hydroalcoholic extract on cardiovascular responses in normotensive rats**

In normotensive animals, 12 mg/kg of the extract significantly decreased SBP and MAP (p<0.05) while, 24 mg/kg significantly increased HR (p<0.001) with no significant effect on SBP and MAP (Table 1).

**Effects of AngII and Losartan on cardiovascular responses**

**Figure 2:**
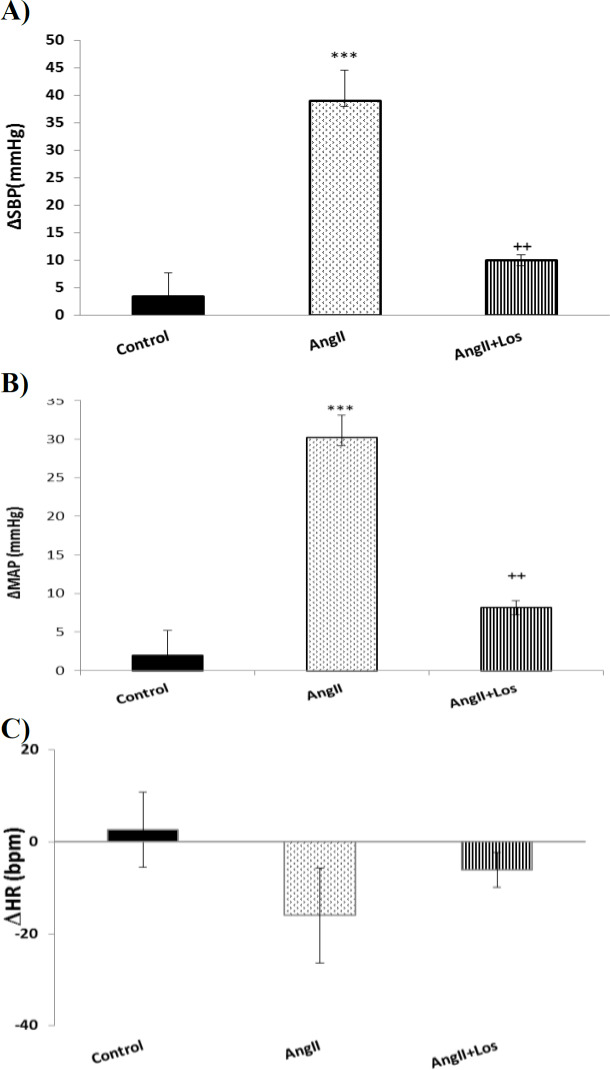
The effects of AngII on ΔSBP (A), ΔMAP (B) and ΔHR (C) in anesthetized rats. Data were expressed as mean ± SEM. One-way ANOVA followed by the Tukey’s post hoc test was used for statistical analysis. (n=10) ***p<0.001 compared to control, ++p<0.01 compare to AngII group

Intravenous injection of AngII significantly increased MAP and SBP with no significant effect on HR. Maximal changes (Δ) of SBP and MAP after injections of AngII are indicated in Figure 2A, B. As has been shown, in AngII group, maximal ΔSBP and Δ MAP significantly increased compared to the control group (p<0.001) while maximal ΔHR was no significantly different with the control group (Figure 2C). 

In Los group, Los injected 10 min before AngII, it significantly attenuated the effect of AngII on ΔSBP and ΔMAP (Figure 2 A, B), (p<0.01) but did not affect ΔHR (Figure 2C).

**The effect of** ***R. khorasanicum*** **hydroalcoholic extract on the cardiovascular responses induced by AngII**

**Figure 3:**
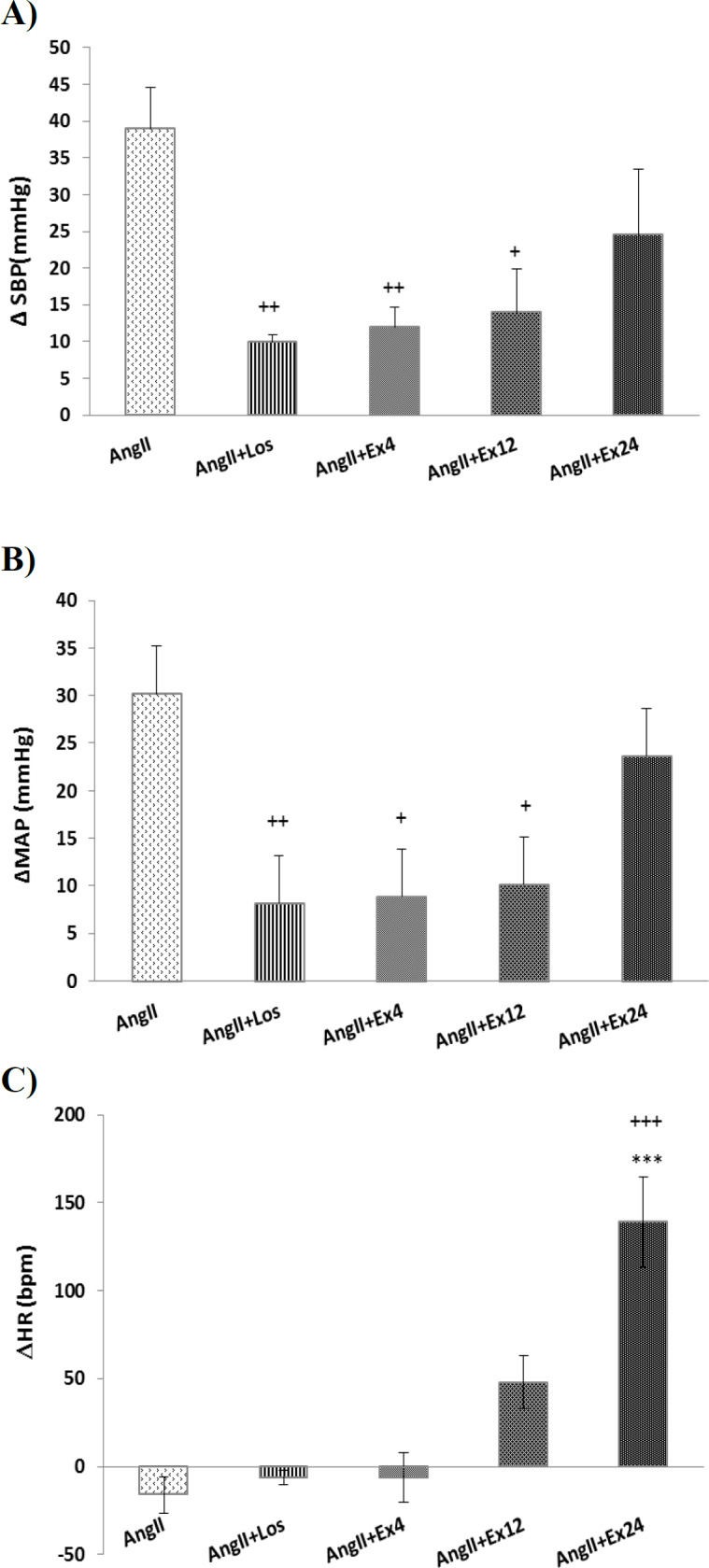
Effects of hydroalcoholic extract of *R. khorasanicum* on the  SBP (A),  MAP (B) and  HR (C) in anesthetized rats. Data were expressed as mean ± SEM. One-way ANOVA followed by the Tukey's post hoc test is used for Statistical analysis. (n=10) +p<0.05, ++p<0.01, +++p<0.001 compared to AngII group

Rats pretreated with three doses of *R. khorasanicum* (4, 12, and 24 mg/kg, i.p). After 30 min, AngII injected and blood pressure and HR were recorded. Dose 4 mg/kg *R. khorasanicum* alone did not significantly affects both ΔSBP and ΔMAP but when administrated before AngII it significantly attenuates both ΔSBP and ΔMAP induced by AngII (p<0.01 and p<0.05, respectively; Figure 3A, B). Dose 12 mg/kg, *R. khorasanicum* reduced ΔMAP and ΔSBP induced by AngII and these changes were significant in respect to AngII alone (p<0.05 for both parameters, Figure 3A, B). 

Dose 12 mg/kg of the extract increased HR compared to pre-injection but this effect did not significantly change in the presence of AngII (p>0.05, Figure 3C).

Dose 24 mg/kg, *R. khorasanicum* did not significantly attenuate the effect of AngII on ΔSBP and ΔMAP; p>0.05, Figure 3A, B). This dose of *R. khorasanicum* alone increased HR and this effect was not affected by AngII (Figure 3C).

**Effect of** ***R. khorasanicum*** **on oxidative stress parameters in the heart and aorta tissues of AngII hypertensive rats**

**Figure 4:**
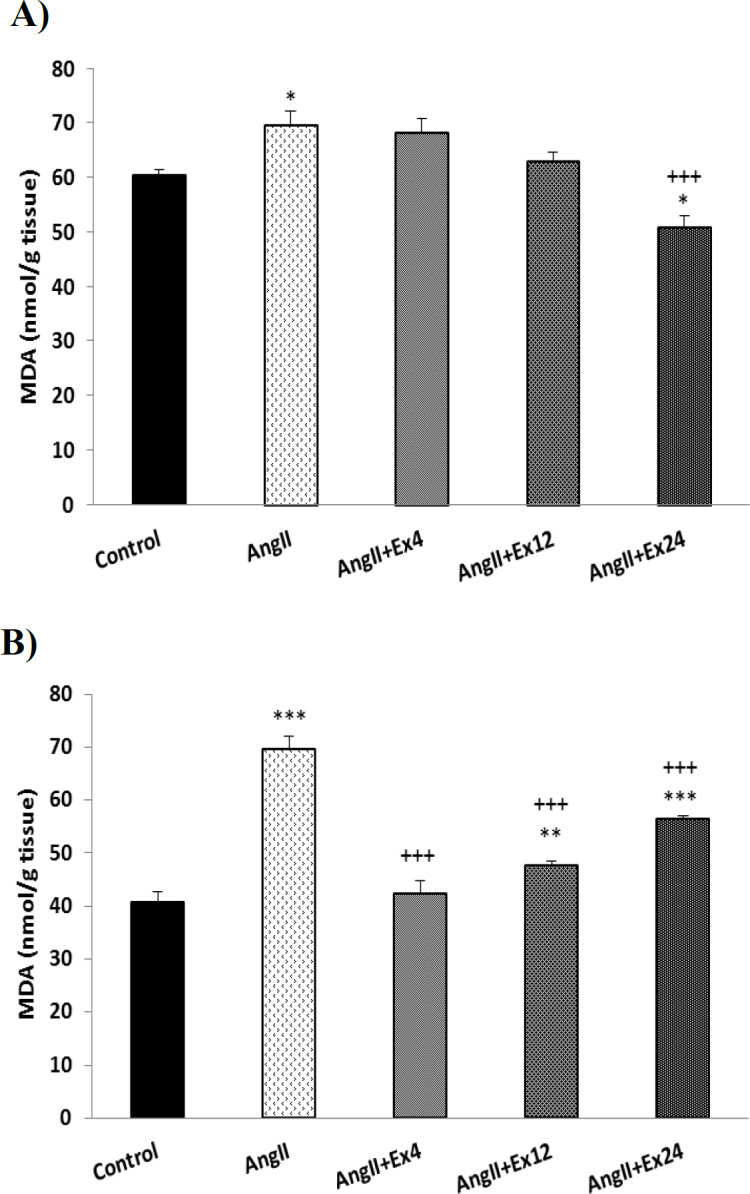
Comparison of the MDA concentration in the heart (A) and aorta (B) tissues. Data are presented as Mean±SEM (n=10 in each group). *p<0.05, **p<0.01 and ***p<0.001 compared to Control group. +++p<0.001 compared to AngII group

In the AngII group, malondialdehyde (MDA) level in heart and aorta tissues significantly increased compared to the saline group (p<0.05 and p<0.001, respectively). In heart tissue only dose 24 mg/kg and in the aorta, all doses of the extract significantly decreased the MDA level compared to AngII group (p<0.001, Figure 4). Total thiol in AngII group, in heart tissue, significantly decreased but in the aorta, the tissue did not significantly change. 

**Table1:** The effect of hydroalcoholic extract of *Ribes khorasanicum* on blood pressure and heart rate in normotensive rats

Groups	**Control**	**EX4 mg**	**EX12 mg**	**EX24 mg**
**Parameter**				
**SBP (mmHg)**	3.4±4.2	-9.1±3.5	-17±3.3 *	-10.9 ±4.5
**MAP (mmHg)**	2.0±3.2	-8.5±3.0	-15.1±2.8*	-7.4±3.9
**HR (bpm)**	2.7±8.1	3.1±9.9	6.8±7.6	117.4±21.9***

Data were expressed as mean±SEM. One-way ANOVA followed by the Tukey’s post hoc test was used for statistical analysis. (n=10) *p<0.05 and ***p<0.001 compared to control.

Bpm: Beat per minute, SBP: systolic blood pressure, MAP: mean arterial pressure

**Figure 5:**
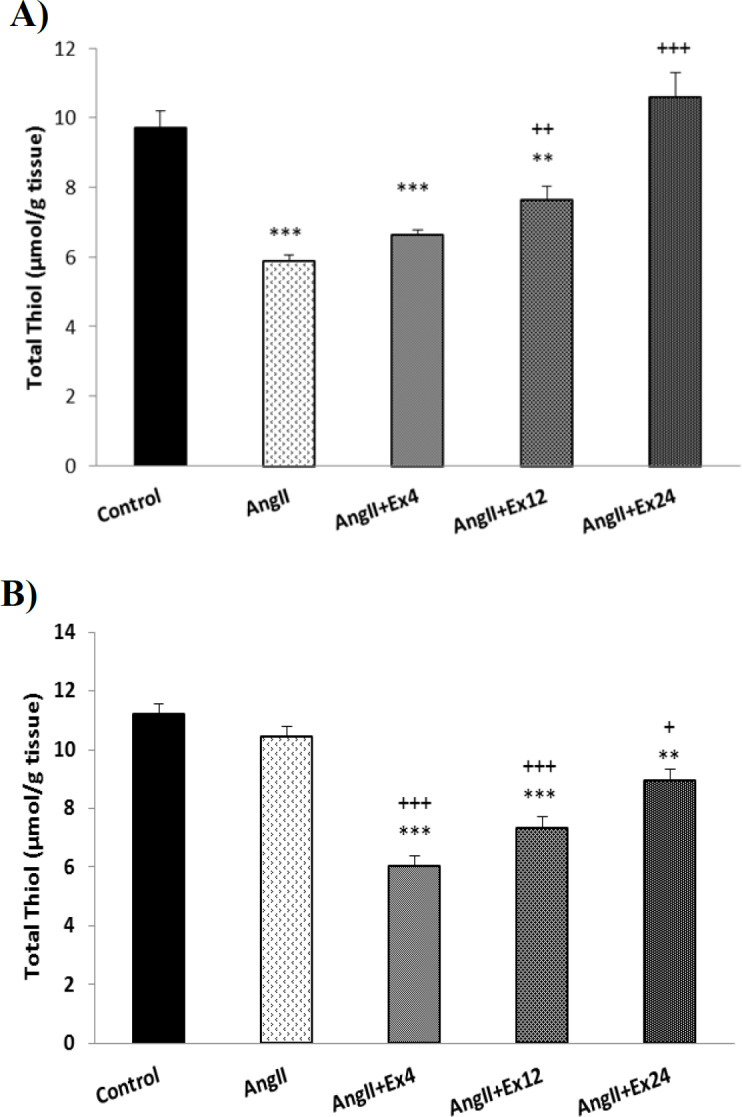
Comparison of total thiol content in the heart (A) and aorta (B) tissues. Data are presented as Mean±SEM (n=10 in each group). **p<0.01 and ***p<0.001 compared to Co group. +p<0.05, ++p<0.01 and +++p<0.001 compared to AngII group

In heart tissue, two higher doses of extract (12 and 24 mg/kg) significantly increased total thiol compared to AngII group (p<0.01 to p<0.001). In aorta tissue also all three doses of the extract significantly increased the total thiol content compared to AngII group (p<0.05 to p<0.001, Figure 5).

**Figure 6:**
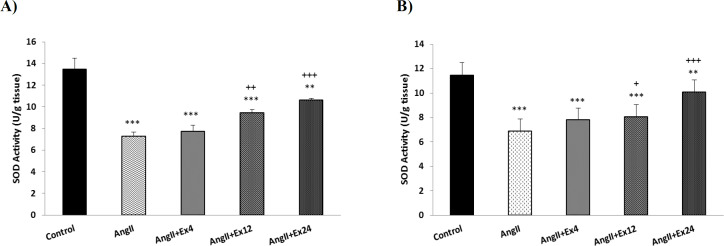
Comparison of SOD activity in the heart (A) and aorta (B) tissues. Data are presented as Mean±SEM (n=10 in each group). **p<0.01 and ***p<0.001 compared to control group. +p<0.05, ++p<0.01 and +++p<0.001 compared to AngII group

The activity of superoxide dismutase enzyme (SOD) in the heart and aorta in AngII group significantly reduced compared to control group (p<0.001) and doses 12 and 24 mg/kg significantly increased SOD level in heart and aorta compared to AngII group (p<0.05 to p<0.001) (Figure 6).

**Figure 7:**
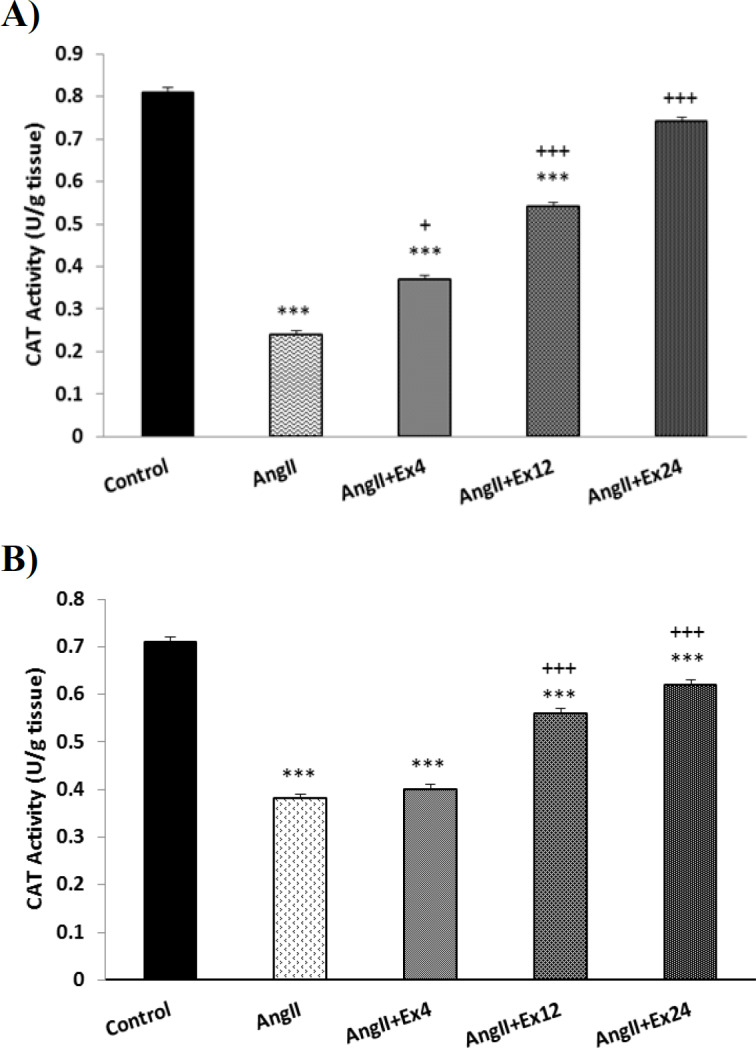
Comparison of CAT activity in the heart (A) and aorta (B) tissues. Data are presented as Mean ± SEM (n=10 in each group). ***p<0.001 compared to Control group. +p<0.05 and +++p<0.001 compared to AngII group

The activity of catalase enzyme (CAT) in the heart and aorta in AngII group significantly reduced compared to the control group. Extract with three doses significantly increased CAT in the heart (p<0.05 to p<0.001) while extract doses, 12 and 24 mg/kg significantly increased CAT activity in the aorta compared to AngII group (p<0.001) (Figure 7).

## Discussion

In the current experiment, the effect of *R. Khorasanicum* on cardiovascular responses in normotensive and hypertension induced by AngII was examined. In normotensive condition, 4 mg/kg of the extract did not significantly affect cardiovascular parameters. However, 12 mg/kg of the extract significantly reduced  MAP and  SBP but  HR did not significantly changed. The higher dose (24 mg/kg) was also significantly increased  HR without significant effect on blood pressure. 

There is little information available about the pharmacologic effect of *R. Khorasanicum*. Primarily phytochemical studies indicated the presence of several compounds such as phenolic compounds especially flavonoids, anthocyanin, saponin, and tannin in this plant. Therefore, each one of these compounds may be contributed in its cardiovascular effect. 

Flavonoids are an important compound of *R. Khorasanicum* fruit that has several effects on cardiovascular system including vasodilation by increased potassium conductance (Calderone et al., 2004 ▶), inhibition calcium inward (Jouad et al., 2001 ▶), increased NO bioavailability and antioxidant effect. Other compounds of the plant are anthocyanin and saponin that have several beneficial effects on cardiovascular system including Ca^2+^ channel blocking and antioxidant property (Rodrigues et al., 2005 ▶; Yuan et al., 2010 ▶). Therefore, each one of these mechanisms may be involved in cardiovascular effect of the extract. 

 The mechanism of plant in higher dose on tachycardia is unknown and we suggest that there is compound(s) that has a vigorous effect on HR, so that doses higher than 100 mg/kg extremely increased HR (unpublished data). Ziberna et al. (2010) ▶ reported that anthocyanin in ischemia-reperfusion injury of isolated heart has a biphasic concentration-dependent effect (Ziberna et al., 2010 ▶). Its low concentration has a protective effect and in higher dose has a toxic effect. Because *R. Khorasanicum* is rich in anthocyanin, it is conceivable that anthocyanin of *R. Khorasanicum* also has a dual effect on heart, and its higher dose increased heart rate via mechanisms such as inhibition of parasympathetic system (Botelho et al., 2017 ▶). Based on this opinion; the hypotensive effect of the extract could be attributed to its vasorelaxant effect (Ziberna et al., 2010 ▶). However, in higher dose, in addition to the vasorelaxation, extract also may directly increase HR (Oliveira et al., 2012 ▶). As a result, the hypotensive effect of the extract is compensated by tachycardia and blood pressure did not drop. However, future experiments are needed to confirm our opinion. 

In the next part of the study, for further investigation of the effect of the extract on the cardiovascular system, its effect on acute hypertension induced by AngII was examined. Our results indicated that low doses of the extract significantly attenuated the effect of AngII on blood pressure with no significant effect on HR and this effect is comparable with Los. However, higher extract dose did not attenuate the effect of AngII. This study suggested that the cardiovascular effect of two lower doses of the extract may be mediated via AngII.

Studies have shown that acute peripheral vasoconstriction is the main effect of AngII which is mediated by angiotensin type 1 receptors (AT1R). Binding of AngII to AT1R elicit several cellular signaling pathways such as phospholipase C and phosphatidylinositol that could enhance intracellular free calcium concentrations, causing vasoconstriction and increased blood pressure (Ferrario, 2006 ▶). 

Ameliorative effect of *R. Khorasanicum* on cardiovascular responses induced by AngII is due to several constituents of this plant. Flavonoids are important constituents of *R. Khorasanicum* (Adibi et al., 2007 ▶; Yazdi et al., 2018b ▶) that have an inhibitory effect on AngII (Apostolidis et al., 2006 ▶; Xue et al., 2008 ▶). Therefore, the antihypertensive effect of this plant may be mediated by this compound. 

Another compound of* R. Khorasanicum* is anthocyanin (Yazdi et al., 2018b ▶)which showed inhibitory effect on the RAS system. Because this compound is highest in this plant (Yazdi et al., 2018b ▶), the effect of *R. Khorasanicum* on AngII is probably due to anthocyanin. However, future studies are needed to clarify our opinion. *R. khorasanicum *also contain saponin with numerous biological effects, including lowering blood pressure. Jeon et al. have shown that saponins is associated with lowering blood pressure in Gold Blatt hypertension (Jeon et al., 2000 ▶). In this study, the antihypertensive effect of the plant can be attributed to the saponins. Effect of the extract on cardiovascular parameters is comparable with Los; therefore, it is possible that the hypotensive effect of extract mediated through inhibition a pathway similar to that of AngII. In higher dose group (24 mg/kg+AngII), pressor effect induced by AngII did not significantly attenuate by the extract. Also, tachycardia induced by the extract was the same as normotensive condition also did not change by AngII. These results indicate that the cardiovascular effect of the extract in this dose is independent to AngII. The mechanism(s) of this effect of the extract was not examined in this study. However, as previously mentioned this effect could be attributed to dual anthocyanin effects of the extract (Ziberna et al., 2010 ▶). 

In this investigation, the effect of the plant extract on oxidative stress parameters also was evaluated. Several pieces of evidence showed that increased oxidative stress in vessels and heart play an important role in the development of hypertension (Romero et al., 1999 ▶). AngII also by increasing superoxide level in endothelial result in endothelial dysfunction and therefore reduce nitric oxide (NO) bioavailability (Usui et al., 1999 ▶). Our results showed that AngII increased the MDA level in the heart and aorta tissues and decreased the tissue level of the total thiol content, the activity of the SOD and CAT enzymes, which is in line with previous studies (Polizio et al., 2008 ▶; Polizio et al., 2005 ▶). The extract of *R. khorasanicum *significantly decreased the effect of AngII on MDA and also increased the level of total thiol content, the activity of SOD and CAT enzymes in cardiac and aortic tissues of treated rats that confirmed the antioxidant role of *R. khorasanicum* extract. The mechanism of the antioxidant effect of the plant is unknown, but in the study of Adibi and Yazdi, this effect of the extract attributed to flavonoids, saponins, alkaloids and also anthocyanin of *R. khorasanicum *(Adibi et al., 2007 ▶). These antioxidant effects could increase NO bioavailability and decreased blood pressure by improvement endothelial dysfunction. 

In summary, this study is a preliminary experiment that shows an inhibitory effect of lower doses of hydroalcoholic extract of R.* Khorasanicum* fruit on blood pressure in normotensive rats. In hypertensive rats, the extract also attenuated cardiovascular responses concomitant with antioxidant effect that is comparable with Los, which suggest that the antihypertensive effect of extract may partly mediated through inhibition of a pathway similar to that of AngII. However, future *in vivo* and *in vitro* studies are needed to better understand the mechanism(s) of cardiovascular effect of this plant. 
